# Characteristics and Educational Support Resources Available to Emergency Medicine Core Faculty: A National Survey

**DOI:** 10.5811/westjem.42503

**Published:** 2025-09-02

**Authors:** Jaime Jordan, Laura R. Hopson, Fiona Gallahue, James A. Cranford, John C. Burkhardt, Keith E. Kocher, Drew L. Robinett, Moshe Weizberg, Tiffany Murano

**Affiliations:** * Oregon Health & Science University, Department of Emergency Medicine, Portland, Oregon; † David Geffen School of Medicine at University of California Los Angeles, Department of Emergency Medicine, Los Angeles, California; ‡ University of Michigan Medical School, Department of Emergency Medicine, Ann Arbor, Michigan; § University of Washington, Department of Emergency Medicine, Seattle, Washington; ¶ Columbia University, Department of Emergency Medicine, New York, New York; || University of Michigan Medical School, Department of Learning Health Sciences, Ann Arbor, Michigan; # Maimonides Midwood Community Hospital, Department of Emergency Medicine, Brooklyn, New York

## Abstract

**Introduction:**

Core faculty are key to supporting the educational mission in emergency medicine (EM). Changes in the Accreditation Council for Graduate Medical Education (ACGME) requirements for minimum protected time for core faculty may no longer guarantee adequate support. We sought to assess EM core faculty characteristics, support, and the impact of the 2019 revisions to ACGME regulations. We explored the influence of individual and institutional characteristics on support and the impact of the regulatory changes.

**Methods:**

This was a cross-sectional survey study of a convenience sample of EM core faculty. Participants completed an online survey of multiple-choice and completion items between April–June 2022. We calculated descriptive and comparative statistics to assess associations between individual (e.g., sociodemographics, rank) and institutional (e.g., region, program type) factors on resources and impact of ACGME revisions.

**Results:**

A total of 596 individuals (57% male) from 116 residency programs participated, including 15 (3%) instructors/lecturers, 280 (47%) assistant professors, 182 (31%) associate professors, and 80 (13%) professors. Most (64%) were 36–50 years of age; 246 (41%) had completed a fellowship. Despite the change to the ACGME requirements in 2019, 417 (70%) reported no modification to their clinical work hours, and 420 (71%) reported no modification to their non-clinical responsibilities. There were statistically significant associations between number of residents per class (*P* < .001), duration of training program (*P* < .001), and type of institution (*P* < .001) with the number of administrative personnel. We also observed statistically significant associations between academic rank (*P* = .02), region (*P* =.01), number of residents per class (*P* = 0.02), and type of site (*P* = .01) with change to clinical work hours after changes to ACGME requirements.

**Conclusion:**

A minority of participants reported a change to their clinical and non-clinical expectations after revisions to the ACGME regulations with disproportionate impact across faculty and program type.

## INTRODUCTION

Academic emergency physicians play a unique and valuable role in the US healthcare system. Although academic emergency departments (ED) make up ~2% of all US EDs, these centers provide care for 5–12% of all acute care patients (> 10 million annually), staffing ~20% of all trauma centers and ~25% of transplant centers.^1^,^2^ However, in addition to their complex patient care responsibilities, the core faculty of these academic centers are charged with multiple extra-clinical responsibilities: training residents and medical students; publishing scholarly work; and filling administrative and quality improvement positions both within and outside the hospital.^3^

Success in these multifaceted roles requires substantial investment in personnel, funds, education, and opportunity.^4^ But, as of 2019, such support may not be guaranteed. The Accreditation Council for Graduate Medical Education (ACGME) changed prior regulations on protected time for CF from a limit on clinical hours to a minimum percentage of support, potentially reducing the administrative and financial support they receive for extra clinical responsibilities of their job.^5^,^6^ This recent change has renewed a century-old discussion on the intrinsic value of academic faculty and how best to support and compensate their work.^7^,^8^ Researchers have investigated the characteristics of this complex issue related to academic faculty roles and support, but often from a top-down perspective in which they summatively assess departments through the responses of program directors or department chairs.^1^,^9^,^10^

To more deeply understand the core-faculty workforce and the resources they are provided to accomplish their critical responsibilities, the field would benefit from data reported directly by the core faculty themselves. In this study, we aimed to characterize this workforce including sociodemographics, roles, responsibilities, administrative support, protected time, and impact of ACGME regulations. We also sought to test the association of these sociodemographic and institutional characteristics on administrative time and funding resources. Understanding these relationships is crucial to informing regulatory bodies and institutional leadership to provide necessary resources and staffing systems that allow faculty to meet the demands of their job tasks and thrive in their uniquely multidimensional roles.

## METHODS

### Design, Setting and Participants

This is a cross-sectional electronic survey study of a convenience sample of core faculty in emergency medicine (EM). We included individuals who were reported as core faculty to the ACGME. We announced the study and directly recruited participants at the Council of Residency Directors in Emergency Medicine (CORD) 2021 Academic Assembly and through emails on the organizational listserv. We also directly reached out to programs to seek diverse representation with regard to region, duration of training, and institution type. We collected data between April–June 2022.

**Population Health Research Capsule**

What do we already know about this issue?

*Core faculty are essential to the educational mission in EM but may not get adequate support to carry out their tasks*.

What was the research question?

*What support do core faculty receive, and how have they been impacted by changes in regulatory requirements re protected time?*

What was the major finding of the study?

*Approximately 70% of participants reported no change to their clinical work hours or non-clinical responsibilities after regulatory revisions*.

How does this improve population health?

*Insights from core faculty themselves on the impact of fewer protected hours illuminate potential downstream impact on teaching, publishing, and fulfilling administrative duties*.

### Study Protocol

We emailed participants a link to an online survey. Informed consent was implied by those who clicked the survey link. We sent up to three reminders to non-responders at regular intervals. We provided participants with a $10 gift card for survey completion. To maximize response rates and minimize guessing, we did not require participants to answer all items on the survey.

### Instrument Development

Our study team of expert educators and education researchers developed the survey after literature review to optimize content validity. We developed the surveys according to best practices in survey design.^11^ The survey consisted of multiple-choice and completion items. We read all items aloud among the author group and piloted the survey with a small group of EM faculty to ensure response process validity. We made revisions for clarity and readability based on feedback. The final survey is available in Appendix A.

### Data Analysis

As this was an exploratory study, we did not conduct statistical power analyses or sample size estimates. We calculated descriptive statistics including percentages and measures of central tendency to detail respondent demographics and responses to survey items with discrete answer choices. We used chi-squared tests, independent-groups *t*-tests, and correlational analyses to examine associations between individual and institutional characteristics with outcome variables of number of administrative personnel, job responsibilities, clinical work hours, and non-clinical expectations. An alpha level of .05 was used for all analyses, and all statistical significance tests were two-tailed. We conducted all analyses with the SPSS software package v29.0 (IBM Corp, Armonk NY).

### Institutional Review Board Statement

This study was reviewed by the Institutional Review Board of the University of Michigan and determined to be “exempt” based on federal exemption category 3(i)(B) at 45 CFR 46.104(d).

## RESULTS

A total of 596 core faculty from 116 EM residency programs participated in this study. We report the characteristics of participants, programs, and institutions in Table 1. Participants were most motivated to be core faculty by the additional opportunities to mentor and teach trainees, to participate in the educational program, and obtain recognition of their educational work with 475 (80.0%), 429 (72.0%), and 261(44.0%) identifying these as one of their top three most important motivators, respectively. While participants received multiple benefits from being core faculty, they had additional responsibilities (Table 2). They found scholarship requirements, completion of assessments, and involvement in the didactic curriculum to be their most challenging responsibilities, with 336 (68.7%), 298 (60.9%), and 238 (48.7%) ranking these as their top three most difficult responsibilities, respectively.After the change to the ACGME requirements in 2020, 417 core faculty (70%) reported no change to their clinical work hours and 420 (70.5%) reported no change to their non-clinical responsibilities (Table 2). Of the 52 participants (11.1%) who reported that the change in ACGME requirements affected their clinical work hours, a greater percentage of assistant (11.3%) and associate professors (11.5%) were affected compared to professors (4.0 %) and instructors/lecturers (0%), *P* = .01. The average number of residents per class was statistically significantly lower among those who indicated that the change in ACGME requirements of July 2020 affected their clinical work hours (mean 11.1 ± 3.3) vs those who indicated that it did not (mean 12.1 ± 3.5), *P* = .02. Type of site was statistically significantly associated with change to clinical work hours after changes to ACGME requirements (P = .01) with 66.7% of military/Veterans Administration (VA) sites, 14.8% of community sites, 9.2% of county/public sites, 8.8% of university sites, and 7.1% of other sites reporting a change to clinical hours. Region was also statistically significantly associated with change to clinical work hours after changes to ACGME requirements (*P* = .09) with 18.0% of programs in the South, 11.7% of programs in the Midwest, 7.5% of programs in the Northeast, and 5.8% of programs in the West reporting a change to clinical hours.Of the 596 study participants, 400 (71.8%) reported that the previous ACGME requirements accurately reflected their commitments and responsibilities. Academic rank was statistically significantly associated with accurate reflection of responsibilities in previous ACGME requirements (*P* = .18) with 84.4% of professors, 85.4% of associate professors, 76.3% of assistant professors, and 62.5% of instructors/lecturers reporting that that the prior ACGME requirements accurately reflected their commitments and responsibilities. The average number of residents per class was statistically significantly higher among those who indicated that the previous ACGME requirements accurately reflected their commitments and responsibilities (mean 12.1 ± 3.4) vs those who indicated that the previous ACGME requirements did not accurately reflect their commitments and responsibilities (mean 11.2 ± 3.5), *P* = .02. There were no statistically significant differences between sex, race, academic rank, type of institution, region, residency duration, or number of residents per class on changes to non-clinical expectations after revisions to the ACGME requirements.

There were statistically significant differences by program duration (three vs four years) and number of residents and number of personnel in program administration. The average number of personnel working in program administration was higher among participants from four-year programs (mean 5.0 [SD = 6.2]) compared to participants from three-year programs (3.1 [SD = 2.6]), *P* < .001. Programs with more residents also had more personnel in program administration (*r*(575) =.18, *P* < .001). The mean number of administrative personnel was also higher in county/public (4.5 [SD = 5.7]) and university (4.4 [SD = 4.3]) than community (2.4 SD = 2.0), military/VA (2.0 [SD = 0.0]) and other (2.8 [SD = 1.8]) training sites (*P* < .001) and higher in the West (4.6 [SD = 5.8]) and Midwest (4.0 [SD = 4.5]) than the South (3.3 [SD = 2.1]) and Northeast (2.7 [SD = 2.3]) regions (*P* < .001).

## DISCUSSION

The previous EM program requirements had a 28 hours/week ceiling on the amount of clinical time that core faculty were permitted to work.^5^ When considering a 40-hour work week, this allotted core faculty 12 hours per week for administration and educational activities. The 2023 requirements establish a floor of 0.1 full-time equivalent (FTE) of protected time for core faculty, or approximately four hours per week in the 40-hour work week model.^15^ Understanding the workforce composition, its responsibilities, and impact of the ACGME changes is critical to determining whether this model of support is adequate. Drawing from a broad cross-section of EM core faculty across geographic regions, program types, and training sites, we are able to describe the core faculty workforce. In comparison to other recent studies of EM, residency core faculty have similar sex distributions to large studies of national specialty organizations.^16^

Our study noted significant associations between academic rank and faculty responsibilities as well as clinical work hours. Most faculty indicated that the prior ACGME requirements accurately reflected their educational commitments, particularly those at the rank of professor and associate professor. These findings may reflect the solidification of responsibilities and alignment with regulatory requirements as faculty progress in their careers. Although at the time of data collection, only a small subset of participants (11%) had been impacted by higher clinical work hours, we found that faculty at the assistant or associate professor rank may be disproportionately affected. These mid-career faculty may have been at the sweet spot to squeeze. They have advanced beyond the very early career stage and may have some administrative time to lose in favor of clinical work compared to clinical instructors who may have already been working substantial clinical time that could not be significantly increased. Yet they are not as advanced in their careers as professors who may have more secure means of protected time such as grant funding or advanced leadership positions.

It is not surprising that programs with larger numbers of residents had more program administrative personnel, highlighting the scaled requirement for resources to the size of the programs.^3^,^15^ This is evident in the ACGME requirements regarding the minimum number of program coordinators, which are scaled to increase with the increased size of a program.^3^,^15^ The higher numbers of additional personnel in program administration among four-year programs is likely a reflection of the relative sizes of four-year programs being overall larger.^17^ Similar associations between size and duration of program have been seen with other outcomes.^18^

Interestingly, although there was no change in the clinical expectations for most participants, there were changes in clinical hours associated with faculty from programs with fewer residents with the new program requirements. This may be due to a perception that smaller programs require less time to administer. While this may be true, there is still a significant amount of time required for engaging in other programmatic and education-related activities that take place regardless of the number of residents in a program (eg, attendance at weekly conference, preparation and delivery of didactic sessions, interview/recruitment efforts, medical student mentoring, scholarship efforts). The correlation between programs with fewer residents and faculty who experienced changes in their clinical work hours as well as their commitments and responsibilities suggests that the smaller programs may have less flexibility in redistributing the clinical and administrative workloads when the ACGME requirements were modified. This potentially places a higher burden on these faculty, expecting them to perform more administrative duties with less time to do so. We also detected associations between type of site and region on changes to clinical hours. This may reflect variations in employment models, funding streams, and institutional priorities.^19^

One of the problems with establishing the floor on protected time, rather than capping the clinical time, is that there is wide variability among institutions (and EDs) as to what is considered 1.0 FTE. Although hour ranges are not explicitly detailed in the literature, institutional definitions of an FTE have been noted to vary from 40 to ≈ 60 hours/week based on individual operational needs and expectations. Emergency departments also vary in what is considered a clinical FTE, 32 vs 36 hours/week.^22^–^24^ With this lack of standardization, the change in the protected time requirement left room for interpretation by organizational, institutional, and departmental leadership to mean that the minimum requirement is the only amount of time necessary for core faculty activities.

## LIMITATIONS

This survey-based study was subject to sampling and response bias with those most engaged in educational programming or most impacted by the ACGME changes potentially being more likely to respond. Future surveys of EM core faculty could be strengthened by systematic assessment of potential non-response bias. While our participants only represent a fraction of the total number of core faculty in EM, they do appear to parallel specialty educator demographics.^25^–^28^ Our data cover a broad cross-section of program characteristics; however, the sample may not be completely representative of the whole.

## CONCLUSION

This study highlights potential concerns about the impact of the changed ACGME requirements for core faculty support on the educational environment for EM residency training. Additional work will be needed to track temporal trends, the potential for disproportionate impact among faculty members and programs, the effect on the learning environment, and the quality of residency training.

## Supplementary Information



## Figures and Tables

**Table 1:** Participant, program, and institution characteristics in a survey of emergency medicine core faculty.

	Total N = 596 n (%)
Sex	
Male	338 (57)
Female	243 (41)
Unknown/Missing	15 (3)
Race	
Asian, Native Hawaiian or other Pacific Islander	57 (10)
Black	12 (2)
Hispanic	36 (6)
White, Non-Hispanic	436 (73)
Other	34 (6)
Prefer not to say/Missing	21 (4)
Age	
< 35	92 (15)
36–50	383 (64)
51–65	95 (16)
> 65	12 (2)
Unknown/Missing	14 (2)
Years in practice in emergency medicine	
0–5	119 (20)
6–10	192 (32)
11–15	116 (20)
16–20	59 (10)
21–25	49 (8)
> 26	50 (8)
Unknown/Missing	11 (2)
Years in educational role	
0–5	244 (41)
6–10	169 (28)
11–15	64 (11)
16–20	53 (9)
21–25	24 (4)
> 26	27 (5)
Unknown/Missing	15 (3)
Fellowship training	
No	337 (57)
Yes	246 (41)
Missing	13 (2)
Fellowship completed*	
Administration	7 (3)
Critical care	12 (5)
Education/Medical education	44 (18)
EMS	21 (9)
Hyperbaric medicine/Dive medicine	1 (0)
Pediatrics	26 (11)
Research	17 (7)
Toxicology	27 (11)
Ultrasound	60 (24)
Other	38 (15)
Missing	5 (2)
Advanced degrees	
MD	502 (86)
DO	68 (12)
MA	26 (4)
MHPE	14 (2)
PhD	10 (2)
Other	121 (21)
MBA	0 (0)
EdD	0 (0)
JD	0 (0)
PharmD	0 (0)
Missing	13 (2)
Academic rank	
Instructor/Lecturer	15 (3)
Assistant Professor	280 (47)
Associate Professor	182 (31)
Professor	80 (13)
Other	26 (4)
Unknown/Missing	13 (2)
Administrative roles**	
Program Director	68 (13)
Assistant/Associate Program Director	127 (25)
Clerkship Director	51 (10)
Assistant or Associate Clerkship Director	21 (4)
Fellowship Director	64 (12)
Medical Director or Assistant/Associate Medical Director	67 (13)
EMS Director or Assistant/Associate EMS Director	40 (8)
Ultrasound Director or Assistant/Associate Ultrasound Director	49 (10)
Research Director or Assistant/Associate Research Director	24 (5)
Vice Chair	46 (10)
Chair	23 (4)
Designated Institutional Official	3 (1)
Assistant/Associate Dean	17 (3)
Other	131 (25)
Missing	79 (13)
Institution has specific faculty promotion tracks	
No	201 (34)
Yes	384 (65)
Unknown/Missing	11 (2)
Specific faculty promotion track	
Clinical Administrator	21 (4)
Clinical Educator	275 (46)
Instructional	9 (1)
Research	27 (5)
Other	50 (8)
Unknown/missing	214 (35)
Promotion track with tenure	
No	427 (72)
Yes	81 (14)
Unknown/missing	88 (15)
Region	
Midwest	131 (22)
Northeast	140 (24)
South	172 (29)
West	153 (26)
Program format	
PGY 1–3	414 (70)
PGY 1–4	166 (28)
Type of primary training site	
Community	195 (33)
County/Public	103 (17)
Military/VA	3 (0.5)
University	243 (41)
Other	34 (6)
Faculty employment model of primary training site	
School of Medicine Employee	234 (40)
Direct Hospital Employee	166 (28)
Large Contract group (Covers > 10 EDs)	93 (16)
Small Contract group (Covers ≤ 10 EDs)	16 (3)
Democratic group	24 (4)
Independent Contractor	9 (2)
Other	39 (7)
Unknown/missing	15 (3)
Number of residents per class (mean ± standard deviation)	12 ± 3.5
Number of personnel in program administration (mean ± standard deviation)	3.6 ± 4

MD, Doctor of Medicine; DO, Doctor of Osteopathic Medicine; MA, Master of Arts

*MHPE*, Master of Health Professions Education; *PhD*, Doctor of Philosophy; *MBA*, Master of Business Administration; *EdD*, Doctor of Education; *JD*, Juris Doctor; *PharmD*, Doctor of Pharmacy; *EMS*, emergency medical services.

* Based on *n* = 246 who responded “Yes” to “Have you completed a fellowship training program?”

** Participants could select more than one role.

*PGY*, postgraduate year; *VA*, Veterans Administration; *ED*, emergency department.

**Table 2:** Reported benefits and responsibilities of being core faculty in emergency medicine,

	M (SD) or n (%) Total N = 596
Responsibilities and benefits received as core faculty*	
Additional clinical time with trainees	223 (41%)
Additional didactics	294 (54%)
Additional administrative responsibilities	399 (73%)
Protected time	372 (68%)
Faculty development opportunities	309 (57%)
Additional compensation	137 (25%)
Other	26 (5)
Missing	49 (8%)
Mean percentage of FTE reduction for being core faculty**	32.0 (21.7)
Mean additional CME funds (in dollars per year) for being core faculty^***^	2,078.2 (3,778.9)
Did the previous ACGME requirements accurately reflect your commitments and responsibilities?	
No	102 (17.1%)
Yes	400 (67.1%)
Unknown/Missing	94 (15.8%)
Did the change to the ACGME requirements in July 2019 affect your clinical work hours?	
No	417 (70.0%)
Yes	52 (8.7%)
Unknown/Missing	127 (21.3%)
Did the change to the ACGME requirements in July 2019 affect your non-clinical expectations?	
No	420 (70.5%)
Yes	43 (7.2%)
Unknown/Missing	133 (22.3%)
If your group decreases their current level of support for core faculty in terms of shift numbers or non-clinical expectations, how would it change your willingness to serve as core faculty?	
Significantly decrease	203 (34.1%)
Slightly decrease	162 (27.2%)
No change	177 (29.7%)
Slightly increase	9 (1.5%)
Significantly increase	5 (0.8%)
Missing	40 (6.7%)
In the past two years, which of the following scholarship requirements for core faculty status have you met?*	
Peer-reviewed publications	410 (75%)
Non-peer-reviewed publications	291 (54%)
Textbooks/chapters	219 (40%)
Presentations at Local/ Regional/National organizations	454 (84%)
Committee leadership	397 (73%)
Editorial services	223 (41%)
Grants	130 (24%)
Missing	52 (9%)

* Participants could select more than one response.

* Based on responses from n = 53 participants.

** Based on responses from n = 232 participants.

*FTE*, full-time equivalent; *CME*, continuing medical education; *ACGME*, Accreditation Council for Graduate Medical Education.
