# Supplementary material for: Characteristics and Educational Support Resources Available to Emergency Medicine Core Faculty: A National Survey
Source: West J Emerg Med. 2025 Sep 2;26(5):1162–9. doi: 10.5811/westjem.42503 (PMC12591615; doi:10.5811/westjem.42503)
Supplement: Supplementary file 1 [file wjem-26-1162-s001.docx]

Appendix A. Survey Instrument

Which of the following best describe your gender identity?

Male

Female

Non-binary / third gender

Prefer to self describe as:

Prefer not to say

Which of the following best describe your racial/ethnic identity?

American Indian or Alaskan Native

Asian

Black/African American

Hispanic

Native Hawaiian or Other Pacific Islander

White, Non-Hispanic

Multi-racial

Other description not listed above

Prefer not to say

What is your age in years?

<35

36-50

51-65

>65

How many years have you been practicing emergency medicine (since Residency completion)?

0-5

6-10

11-15

16-20

21-25

>26

How many years have you been in an educational role (PD, APD, CD, Core Faculty Member, etc.)

0-5

6-10

11-15

16-20

21-25

>26

Please select the academic rank that best describes your current appointment or academic rank.

Professor

Associate Professor

Assistant Professor

Instructor/Lecturer

Other : __________________________________________________

Are you on a faculty track that grants tenure at your institution?

No

Yes

Don't Know

Does your institution have specific faculty tracks and do you know which you are appointed to?

No

Yes

Which of the following best describes the faculty track you are on?

Clinical Educator

Instructional

Research

Clinical Administrator

Other : __________________________________________________

What Advanced Degrees do you hold (Please select all that apply)?

MD

DO

MA

MHPE

MBA

PhD

EdD

JD

PharmD

Other :__________________________________________________

Have you completed a fellowship training program?

No

Yes

What was your fellowship in (Please select all that apply)?

Education/Medical Education

EMS

Research

Adminstration

Ultrasound

Pediatrics

Critical Care

Hyperbaric Medicine/Dive Medicine

Toxicology

Other :__________________________________________________

In addition to your role as core faculty, do you have any additional administrative roles or titles within the department or medical school (Please select all that apply)?

Clerkship Director

Associate or Assistant Clerkship Director

Program Director

Associate or Assistant Program Director

Fellowship Director

Medical Director or Associate/Assistant Medical Director

EMS Director or Associate/Assistant EMS Director

Ultrasound Director or Associate/Assistant Ultrasound Director

Research Director or Associate/Assistant Research Director

Vice Chair

Chair

DIO (Designated Institutional Official)

Dean

Associate or Assistant Dean

Other : __________________________________________________

What is the duration of your core EM residency program?

3 years

4 years

How many residents in each entering class?

________________________________________________________________

Which of the following best describes the faculty employment model of your primary training site for the residency?

School of Medicine Employees

Direct Hospital Employee

Large Contract Group (Covers >10 EDs)

Small Contract Group (Covers 10 or Fewer EDs)

Democratic Group

Independent Contractors

Other : __________________________________________________

Which of the following best describes your primary training institution for your residency program?

Community

County/Public

Military/VA

University

Other :__________________________________________________

How many additional personnel work in program administration (Coordinators, Administrative Assistants, etc.)?

What are the benefits and responsibilities that you receive as a core faculty status at your institution? (Please check all that apply)

Additional clinical time with trainees

Additional didactics

Additional administrative responsibilities

Protected time

Additional Continuing Medical Education (CME) Funds

Faculty Development opportunities

Additional compensation

Other : __________________________________________________

What percentage of FTE reduction do you receive as a Core Faculty Member?

________________________________________________________________

How much additional funds (in dollars per year) do you receive in CME funds?

________________________________________________________________

The previous ACGME requirements for core faculty include working no more than 28 hours per week on average, or 1344 hours per year, whichever is less. Did these accurately reflect your commitments and responsibilities?

Yes

No

Unsure

Did the change in these requirements of July 2019 affect your clinical work hours?

Yes

No

Unsure

Did the change in these requirements of July 2019 affect your non-clinical expectations?

Yes

No

Unsure

If your group decreases their current level of support for core faculty in terms of shift numbers or nonclinical expectations, how would it change your willingness to serve as core faculty?

Significantly Decrease

Slightly Decrease

No Change

Slightly Increase

Significantly Increase

In the past two years, which of the following scholarship requirements for core faculty status have you met?: (Please check all that apply)

Peer Reviewed Publications

Non-peer Reviewed Publications

Textbooks/chapters

Presentations at Local/Regional/National Organizations

Committee Leadership

Editorial Services

Grants

Please rank the following elements in the order of IMPORTANCE to your motivation in being designated as core faculty (1=most important)

______ Recognition of educational work

______ Additional opportunities to mentor/advise/teach trainees beyond that of other faculty

______ Scholarship

______ Opportunities to participate in the educational program (e.g. didactic program, curriculum design, etc.)

______ Benefits to myself in the clinical environment (e.g. shift reduction, shift schedule, etc.)

______ Benefits of additional faculty development (e.g. access to funds, programmatic opportunities, etc.)

______ Other

Please rank the following core faculty responsibilities in order of DIFFICULTY for you to meet ACGME requirements (1=most difficult)

______ Clinical supervision and teaching

______ Completion of assessments

______ Involvement in didactic curriculum

______ Involvement in curriculum design

______ Regular participation in organized discussion, rounds, journal clubs, and conferences

______ Scholarship requirements

______ Other
